# Enhanced Virulence of *Aeromonas hydrophila* Is Induced by Stress and Serial Passaging in Mice

**DOI:** 10.3390/ani11020508

**Published:** 2021-02-16

**Authors:** Kyoo-Tae Kim, Seung-Hun Lee, Kyoung-Ki Lee, Jee Eun Han, Dongmi Kwak

**Affiliations:** 1 Animal Health Center of Zoo Land, Daejeon O-World Theme Park, Daejeon 35073, Korea; ktkim95@korea.kr; 2 College of Veterinary Medicine, Chungbuk National University, Cheongju 28644, Korea; dvmshlee@chungbuk.ac.kr; 3 Animal Disease Diagnostic Division, Animal and Plant Quarantine Agency, Gimcheon 39660, Korea; naturelkk@korea.kr; 4 College of Veterinary Medicine, Kyungpook National University, Daegu 41566, Korea; jehan@knu.ac.kr; 5 Cardiovascular Research Institute, Kyungpook National University, Daegu 41944, Korea

**Keywords:** aerolysin, *Aeromonas hydrophila*, hemolysin, mice, stress, virulence

## Abstract

**Simple Summary:**

*Aeromonas hydrophila*, which is an opportunistic zoonotic bacterium, has the ability to infect animals with injuries involving the condition of the aquatic environments. Factors including poor sanitation and water quality, stress, overcrowding, and rough handling can make animals more sensitive to infections and trigger outbreaks of *A. hydrophila*. *A. hydrophila* was previously isolated from an African black-footed penguin that died while in captivity at a zoo, following clinical signs of depression and anorexia, and in this study, we investigated the effect of stress and serial passaging in mice on *A. hydrophila* virulence. Serial passaging in mice enhanced the virulence of *A. hydrophila*, and *A. hydrophila* infection combined with administration of stress hormones or fasting increased mortality.

**Abstract:**

*Aeromonas hydrophila* was isolated from an African black-footed penguin (*Spheniscus demersus*) that died while in zoo captivity. At necropsy, the virulence of *A. hydrophila* appeared to be enhanced by stress, so was assessed in the presence of in vitro and in vivo stressors and serial passaging in mice. Virulence genes from the isolate were amplified by PCR. In vitro assays were conducted to test the hemolytic activity, cytotoxicity, and effect of stress hormones on *A. hydrophila* virulence. In vivo assays were conducted to test the stress effect on mortality of *A. hydrophila*-infected mice and virulence in mice. Two virulence genes coding for hemolysin (*ahh1*) and aerolysin (*aerA*) were detected, and the cytotoxic potential of the isolate was demonstrated in baby hamster kidney and Vero cells. Some or all mice inoculated with *A. hydrophila* and exposed to stress hormones (epinephrine and norepinephrine) or low temperature died, while mice inoculated with *A. hydrophila* and exposed to fasting or agitation stressors or no stressors survived. We concluded that stress can be fatal in mice experimentally infected with *A. hydrophila* and that serial passaging in mice dramatically enhances the virulence of *A. hydrophila*.

## 1. Introduction

Members of the genus *Aeromonas* are ubiquitous Gram-negative bacilli that are widespread in aquatic environments [1]. *Aeromonas* spp., as a component of the natural microflora of aquatic bodies, are found in aquatic environments worldwide, including ground, surface, estuarine, marine, waste, and drinking water. Aeromonads are also found in many foods, including fresh produce, seafood, raw meats, packaged ready-to-eat foods, cheese, and milk [2]. Broadly, aeromonads can be divided into motile and non-motile species [3,4]. Several motile *Aeromonas* species (e.g., *A. hydrophila*, *A. caviae*, *A. dhakensis*, and *A. veronii* biovar sobria) are known as pathogens of aquatic animals, and interest in this genus has increased due to its zoonotic potential [3,4].

Motile *Aeromonas* is a common agent of motile *Aeromonas* septicemia, which is also referred to as epizootic ulcerative syndrome. The infection includes tissue swelling, skin ulcers, necrosis, and hemorrhagic septicemia [3,4,5]. Commonly, *Aeromonas* septicemia has been reported in aquatic animals [6,7,8,9]. In humans, *Aeromonas* infection does not show any clinical signs, except in cases of immunosuppression, chronic disease, or trauma, but it can lead to severe complications in some cases [10,11,12]. Moreover, in several freshwater fish both in the farm and field, motile *Aeromonas* spp. are natural gut microbiota and regarded as opportunistic pathogens; the disease is frequently associated with hemorrhagic septicaemia [13,14,15].

The virulence factors of *Aeromonas* spp. are present in two forms: cell-associated structures and extracellular products [16]. Cell-associated structures include pili, flagella, outer membrane proteins, lipopolysaccharide, and capsules. These structural features promote bacterial attachment and protect cells from the host immune response. The major extracellular products include cytotoxic, cytolytic, hemolytic, and enterotoxic proteins [17]. Multiple cytotoxins, aerolysins, hemolysins, and cytotoxic enterotoxins are associated with the virulence of *Aeromonas* spp. in humans and animals. Aerolysin is a characteristic virulence factor of the genus [18,19]. 

Cases of *Aeromonas* infection associated with captivity-induced stress have been reported in aquatic animals, such as spectacled caiman (*Caiman crocodilus*) and European pond turtle (*Emys orbicularis*) [8,20]. In our previous study, *A. hydrophila* was isolated from an African black-footed penguin (*Spheniscus demersus*) that died while in captivity at a zoo, following clinical signs of depression and anorexia with greenish vomitus [7] and we further investigated its virulence in several aspects, such as virulence genes, virulence change in serial passage, and administration of stress hormones or fasting in this study.

## 2. Materials and Methods

### 2.1. Compliance with Ethical Standards

This study was performed in strict accordance with protocols approved by the Institutional Animal Care and Use Committee (approval no. DOTP-200600501) at the Daejeon O-World Theme Park. Animal husbandry, monitoring of health status, and euthanasia criteria were applied as described in the manuscript, and all efforts were made to minimize unnecessary suffering and distress of the animals.

### 2.2. Bacterial Isolates and Reference Strain

From the liver and the intestine of the penguin died while in captivity, a Gram-negative, motile, and rod-shaped bacterium was dominantly isolated by streaking the bacteria on a blood agar plate (Asan Pharmacy, Seoul, Korea). The isolates producing beta hemolysis in the blood agar were identified as *A. hydrophila* by API 20E/NE rapid identification system (BioMérieux, Craponne, France), and then stored in a tryptic soy broth medium (TSB; Difco, Franklin Lakes, NJ, USA) supplemented with 15% (v/v) glycerol at −70 °C [7]. A reference *A. hydrophila* strain (KCTC #2358) was purchased from the Biological Resource Center (Daejeon, Korea). The isolate and reference strains were cultured in TSB at 37 °C for 24 h with shaking. Following centrifugation, the supernatants were filtered using a 0.22-μm membrane filter (Millipore, Billerica, MA, USA). The filtrates were stored at −70 °C until used.

### 2.3. Singleplex and Multiplex PCR for A. hydrophila Identification and Virulence Genes

Bacterial DNA was extracted using a DNA Extraction Kit (Bioneer, Daejeon, Korea) according to the manufacturer’s instructions. Two virulence genes were used as PCR targets, as previously described [15]: the extracellular hemolysin gene (*ahh1*) and aerolysin gene (*aerA*). In addition, a primer set for *A. hydrophila* 16S rRNA, which is a housekeeping gene, was used as an internal control [15].

### 2.4. Hemolytic Activity and Cytotoxicity of the Isolate

The hemolytic activity of the isolate was evaluated using cell-free hemolytic assay with culture supernatants. Briefly, *A. hydrophila* culture supernatants (100 μL) were serially diluted in 100 μL phosphate buffered saline (PBS; pH 7.4) and mixed with an equal volume of a 1% suspension of rabbit erythrocytes in microtiter plates. The plates were incubated at 37 °C for 1 h, followed by further overnight incubation at 4 °C. The hemolytic activity was recorded as the highest serial dilution that displayed hemolysis [21]. Equal volumes of the reference strain and PBS alone were added as controls.

To study the cytotoxicity, Vero and baby hamster kidney (BHK) cells were grown in Eagle’s minimal essential medium with 10% fetal bovine serum (Gibco, Waltham, MA, USA); 2 mM l-glutamine; 100,000 U/L penicillin; and 10 mg/mL streptomycin (Sigma-Aldrich, St. Louis, MO, USA). The cells were incubated in 96-well tissue culture plates at 37 °C in an atmosphere of 5% CO_2_. Confluent monolayers (10^5^ cells/mL) were added to two-fold serial dilutions of bacterial culture supernatants, and the plates were incubated at 37 °C in a humidified atmosphere of 5% CO_2_. The cells were examined using an inverted microscope (Nikon, Tokyo, Japan), and morphological changes in the cells due to *A. hydrophila* were scored following incubation for 0, 3, and 24 h. Briefly, vacuolating effects with total detachment and destruction of the monolayer including cell death were evaluated, and the highest serial dilution that displayed cytotoxicity was recorded as the cytotoxicity titer according to the method described [21]. Equal volumes of the reference strain and PBS alone were added as controls.

### 2.5. Effect of Stress Hormones on the In Vitro Growth of A. hydrophila

To determine the effect of stress hormones on the in vitro growth of *A. hydrophila*, the isolated *A. hydrophila* strain was cultured in TSB at 37 °C for 18 h, and the turbidity was adjusted to match that of 0.5 McFarland standards (1.5 × 10^8^/mL) for inoculation. The hormones norepinephrine and epinephrine (Sigma-Aldrich, St. Louis, MO, USA) were prepared at 0.1 M in PBS, and each solution was filtered through a 0.2-μm membrane (Millipore). These solutions were further diluted 10-fold in PBS to prepare solutions ranging from 10^−2^ to 10^−5^ M [12], which were added to the broth. The growth of *A. hydrophila* was estimated using McFarland standards after incubation for 0, 24, 48, and 72 h at room temperature.

### 2.6. Effect of Stressors on A. hydrophila Virulence

To determine whether stress conditions enhanced the mortality of *A. hydrophila*-infected mice, the effect of epinephrine and norepinephrine stress hormone as well as the physical stressors of fasting, low temperature, and agitation were tested.

Seventy Institute of Cancer Research (ICR) mice (Orient Bio, Seongnam, Korea) with an average age of 6 weeks and body weight of 20–25 g were divided into seven groups, with 10 mice in each group (five males and five females). In the experiment, 1 mL culture suspension (1.5 × 10^8^ *A. hydrophila*) was orally administered to each mouse, except those in the group treated with 1 mL PBS. Then, each group was treated with 1 mL norepinephrine (0.1 M; AH + N), 1 mL epinephrine (0.1 M; AH + P), low temperature-induced stress at 8 °C (AH + L), agitation stress by shaking the cages twice each day at 100 times/min for 2 h (AH + S), fasting providing only tap water (AH + F), *A. hydrophila* alone (AH alone), or PBS without *A. hydrophila* (PBS).

Five mice of the same sex were housed in a standard Plexiglass cage (50 × 25 × 17.5 cm^3^) with temperature (22 °C ± 1 °C) and humidity (55% ± 5%) controlled during a reversed 12-h dark/light cycle, with the light period occurring from 8:00 a.m. to 8:00 p.m. Mice except for the fasting group had ad libitum access to tap water and commercial pellets for laboratory animals without any additives (Woosung Feed Co., Daejeon, Korea). The mice were monitored three times a day for health status, and there were no unexpected deaths. Mice were euthanized if they met any of the criteria for early removal (lethargy, hunched posture, ruffled coat, or difficulty standing) to limit suffering. Anesthetic (sodium pentobarbital) overdose was used for euthanasia. Living and euthanized mice were counted daily for four days.

### 2.7. Necropsy and Histopathology

All living mice were euthanized at the end of the experiment as described above and subjected to necropsy. Tissue samples including the heart, liver, lung, and small intestine were fixed in 10% neutral formalin and then embedded in paraffin. The tissue samples were sectioned and the sections stained with hematoxylin and eosin and laid on HistoBond^®^ slides (Lab Monster, Seoul, Korea). The sections were used for Lillie’s Gram staining for bacterial identification.

### 2.8. In Situ PCR

Tissue sections were mounted on slides, deparaffinized at 60 °C for 18 h, immersed first in xylene at 37 °C for 30 min and then in absolute ethanol, 75% ethanol, 50% ethanol, 25% ethanol, and water at room temperature. The sections were permeabilized by incubation at room temperature in 0.02 mol/L HCl for 10 min, followed by 0.01% Triton X-100 for 90 s proteins were eliminated by incubation with 20 μg/mL protease K (Gibco) at 37 °C for 10 min, which was further inactivated by boiling in a microwave for 15 s, and the slide sections were plunged into 20% acetic acid for 15 s to inactivate endogenous alkaline phosphatase.

PCR was performed by incubating these sections with 50 mL of 1× reaction buffer (Gibco), 5 U Taq polymerase, 2.5 mmol/L MgCl_2_, 2.5 mmol dNTP, 0.4 mmol biotin-14-dCTP, and 100 pmol primers for the 16S rRNA sequence of *A. hydrophila*, as previously described [15]. The slides were sealed using an assembly tool (Takara, Shiga, Japan) and placed in a thermocycler (Perkin Elmer, Waltham, MA, USA). PCR was performed with 25 cycles of denaturation at 95 °C for 1 min, annealing at 59 °C for 1 min, and extension at 72 °C for 1 min. PCR products were detected using diluted (1:100) normal goat serum for blocking and diluted (1:5000) streptavidin horseradish peroxidase conjugate for detection. The chromogen was 3,3’-diaminobenzidine (DAB; Zymed, San Francisco, CA, USA). The slides were counterstained with hematoxylin.

### 2.9. Effect of Serial Passaging on Virulence

To determine whether the virulence of *A. hydrophila* could be enhanced by serial passaging in mice, the *A. hydrophila* isolated from the liver of the dead penguin was streaked on blood agar (Asan Pharmacy, Seoul, Korea) and incubated at 37 °C for 18 h before inoculation into Mueller-Hinton broth (MHB, Difco). The turbidity of the culture suspension was adjusted to 0.5 McFarland standards (1.5 × 10^8^/mL) before inoculation. A total of 30 ICR mice (average age, 6 weeks; body weight, 20–25 g) were used for infection in each passage. For the first passage, the “oral passage” and “intraperitoneal passage” experimental groups each comprised 10 mice, as did the control group. Mice in the experimental groups were inoculated with 1 mL *A. hydrophila* suspension adjusted to 0.5 McFarland standards, via the oral and intraperitoneal routes. Control mice were inoculated with an equal volume of PBS. Then, *A. hydrophila* was re-isolated from the liver of the mice by streaking and incubation at 37 °C for 18 h on blood agar. The bacterial suspension prepared in MHB was inoculated again into mice at the dosage of 1.5 × 10^8^ colony forming unit (CFU) per mouse for the second passage. This step was repeated again for the third passage. Cages, food, water, and bedding were confirmed to be *A. hydrophila*-free before use. Animal husbandry, monitoring of health status, and the criteria for euthanasia were applied as described above, and all efforts were made to reduce the distress and suffering of animals.

After euthanizing the mice, tissue samples were aseptically inoculated onto blood agar (Asan Pharmacy) and MacConkey agar (Difco) and incubated at 37 °C for 12–18 h. Isolates were identified by API-20E or API-20NE (BioMérieux, Craponne, France).

## 3. Results

### 3.1. Singleplex and Multiplex PCR for A. hydrophila Identification and Virulence Genes

**Figure 1:**
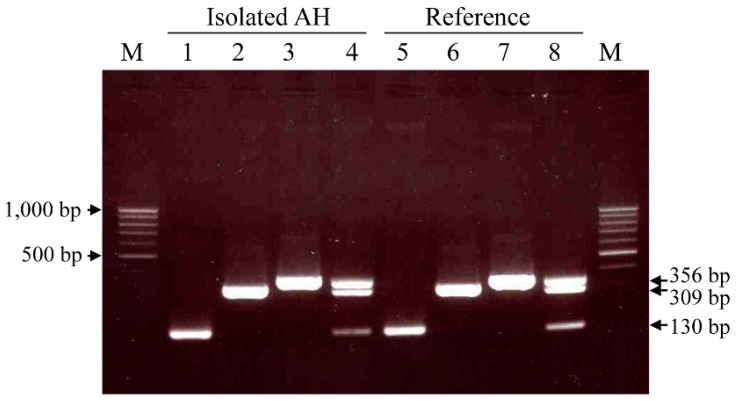
Singleplex and multiplex PCR-based detection of virulence and 16S rRNA genes of *Aeromonas hydrophila* isolated from a dead African black-footed penguin and the reference strain KCTC #2358. Lanes: M, 100 bp ladder; 1 and 5: *ahh1*; 2 and 6: *aerA*; 3 and 7: 16S rRNA; 4 and 8: triplex PCR.

The *ahh1*, *aerA*, and 16S rRNA amplicons were amplified in the *A. hydrophila* isolate and reference strain (Figure 1). Therefore, the isolate carried the virulence genes for hemolysin and aerolysin.

### 3.2. Hemolytic Activity and Cytotoxicity Assay

The hemolytic activity titer of the *A. hydrophila* isolate on rabbit erythrocytes (64) was considerably lower than that of the reference strain (256), while the cytotoxicity titer was the same or higher than those of the reference strain in Vero and BHK cells (Table 1).

### 3.3. Effect of Stress Hormones on the In Vivo Growth of A. hydrophila

**Figure 2:**
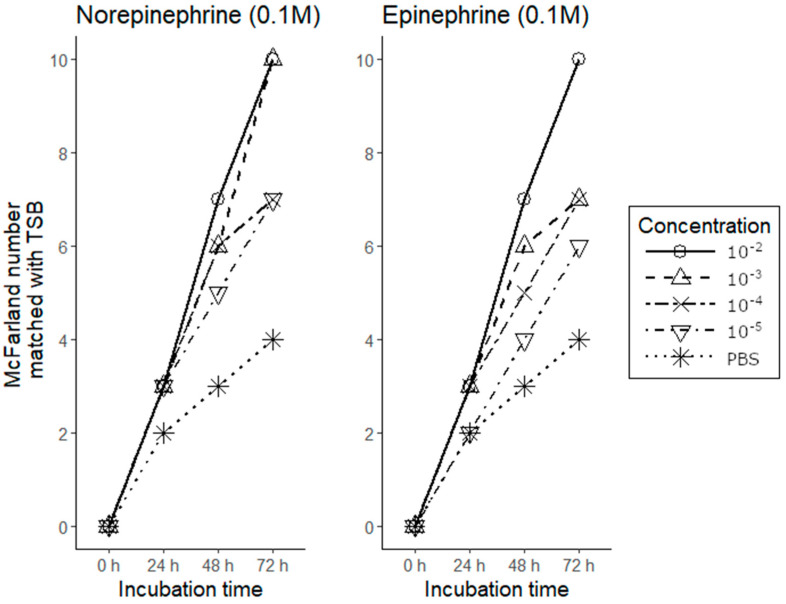
Effect of stress hormones (norepinephrine and epinephrine) on the growth of *Aeromonas hydrophila*. TSB, tryptic soy broth; PBS, phosphate buffered saline.

Norepinephrine treatment led to increased growth of *A. hydrophila* compared to the control, with the effect observed as early as 24 h (Figure 2). Similar results were observed with epinephrine treatment. The extent of *A. hydrophila* growth decreased with dilution of the stress hormones.

### 3.4. Effect of Stressors on A. hydrophila Virulence

In the norepinephrine group, all 10 mice were euthanized within two days of treatment (Table 2). However, in the epinephrine group, only four mice were euthanized. Six mice remained alive at day 4 post-treatment. Concerning physical stresses, all mice in the fasting group died at day 4 post-treatment, but there were no deaths in the low temperature or agitation groups. In addition, *A. hydrophila* alone did not cause any death during the experiment. Notably, *A. hydrophila* was isolated from all euthanized mice, but not from living mice.

### 3.5. Necropsy and Histopathology

**Figure 3:**
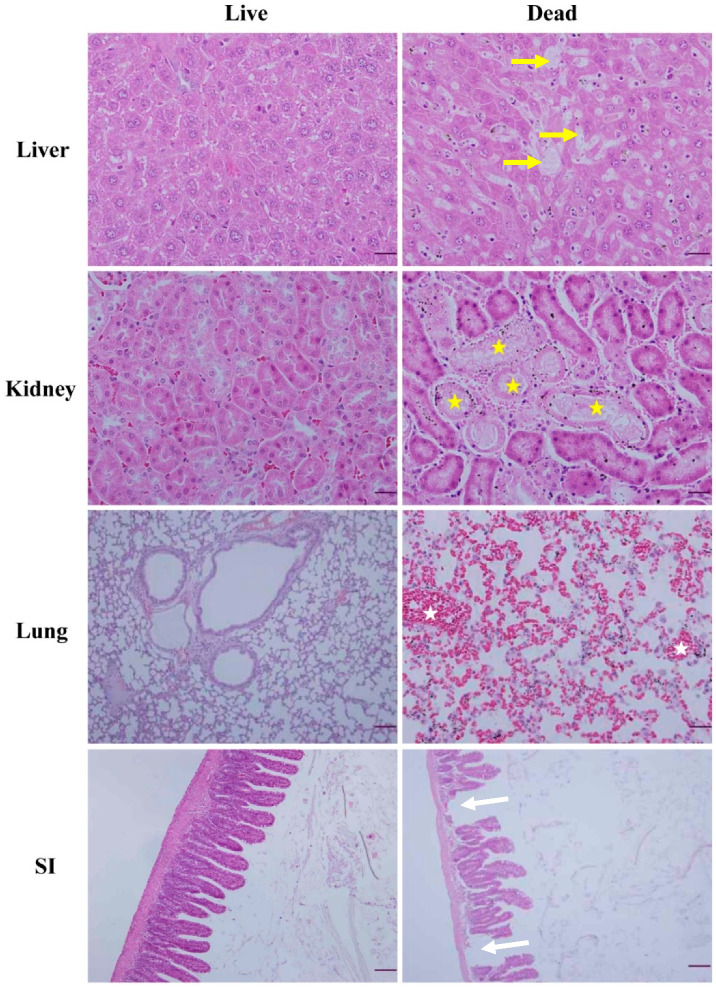
Histopathology of mice infected with *Aeromonas hydrophila*. Tissue samples including those of the liver, kidney, lung, and small intestine (SI) from dead and living mice were stained with hematoxylin and eosin. The living mice were from mice that were infected with *A. hydrophila* but did not die, and dead mice were from mice that died following infection with *A. hydrophila*. Diffuse vacuole degeneration and necrosis of the infected liver (yellow arrows), tubular necrosis of the infected kidney (yellow stars), severe hemorrhagic signs on lung tissue (white stars), and detached necrosis of SI (white arrows) were observed in dead mice. Bars (20 μm; ×400) in liver, kidney, and lung; bars (200 μm; ×40) in SI.

Compared to the tissues of living mice, lesions compatible with severe tissue damage were observed in several organs. The liver displayed diffuse focal necrosis. The kidney displayed renal tubule necrosis. Severe hemorrhagic signs were observed in lung tissue, and obvious detachment of the villi was observed in the small intestine (Figure 3). 

**Figure 4:**
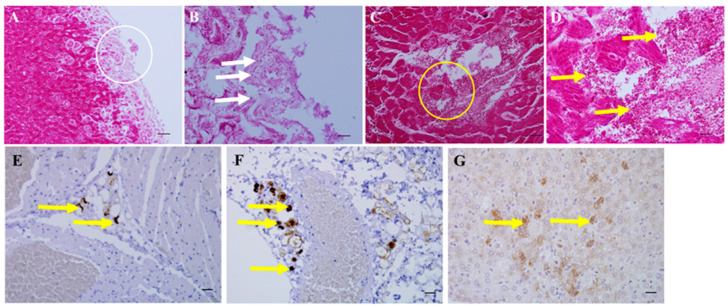
Visualization of *Aeromonas hydrophila* in mouse tissues using the Lillie’s Gram staining (**A**–**D**) and in situ PCR (**E**–**G**). A, bacteria in the liver (bar = 50 μm; ×200); B, magnification of the image in the white circle in panel A and white arrows pointing to bacteria (bar = 10 μm; ×1000); C, bacteria in the heart (bar = 20 μm; ×400); D, magnification of the image in the yellow circle in panel C with bacteria indicated by the yellow arrows (bar = 10 μm; ×1000); E, heart (bar = 20 μm; ×400); F, lung (bar = 20 μm; ×400); and G, liver (bar = 20 μm; ×400). Yellow arrows in E, F, and G indicate positivity of in situ PCR.

Gram staining revealed red and rod-shaped bacteria in liver and heart tissues of the euthanized mice (Figure 4). In situ PCR revealed that the presence of *A. hydrophila* 16S rRNA, which was detected as light to dark brown staining in the heart, lung, and liver tissues of the dead mice (Figure 4).

### 3.6. Effect of Serial Passaging on Virulence

In the oral inoculation group, the first passage resulted in the euthanasia of one mouse 72 h post-inoculation. The second passage resulted in the euthanasia of one mouse each at 48 and 72 h post-inoculation. However, in the third passage, all 10 mice were euthanized within 12 h post-inoculation (Table 3). In the intraperitoneal inoculation group, the first, second, and third passages led to the euthanasia of all mice at 48, 24, and within 12 h post-inoculation, respectively.

During serial passaging, clinical signs were observed. These included diminished consumption of diet, dull behavior, and depression. These signs were more pronounced with intraperitoneal inoculation compared to oral administration. At necropsy, a mildly congested intestine was observed, which was more severe in the intraperitoneal group.

## 4. Discussion

In the previous study [7], the penguin died while in captivity at a zoo, following clinical signs of depression and anorexia with greenish vomitus. At necropsy, dead penguin appeared to have hemorrhage and catarrhal inflammation of the intestines, severe enlargement of the right hepatic lobe, elongation of the gall bladder, and pyloric ulceration of the stomach. Microscopic observation revealed congestion, fat droplets within the cytoplasm of the hepatic cell, infiltration of lymphocytes in the stomach, villus detachment, and destroyed glandular epithelium in the small and large intestines. From the liver and intestine of the dead penguin, *A. hydrophila*, but no other causative agents, was isolated [7].

According to a previous study [22], Proteobacteria including *Aeromonas* species are natural gut microbiota in penguins. However, stress might lead to *Aeromonas* infection in case animals with decreased immune functioning [3,4,5]. In several studies, it was reported that catecholamines (including epinephrine and norepinephrine) increased bacterial growth, virulence-associated factors, adhesions, and biofilm formation and consequently influenced bacterial infections in many hosts [23]. This also has been examined in several aquatic pathogens, including *Aeromonas, Edwardsiella,* and *Vibrio* spp. [12,24,25].

In this study, we investigated the virulence of the *A. hydrophila* isolate in several aspects, such as virulence genes, virulence change in the serial passage (in vivo), and administration of stress hormones (in vitro) or fasting (in vivo). In a previous study [18], 87 *A. hydrophila* isolates of enteric origin all tested positive for *ahh1*, 55% tested positive for *aerA*, and all tested positive for 16S rRNA by multiplex PCR. In the present study, the *A. hydrophila* strain isolated from the dead penguin and the reference strain tested positive for *ahh1*, *aerA*, and 16S rRNA by singleplex and multiplex PCR. When 107 *A. hydrophila* isolates from ready-to-eat meat and cheese in Italy were examined for cytotoxins, all isolates were cytotoxic to Vero cells [21]. Examination of 48 *A. hydrophila* isolates from seafood detected 79.2% hemolysin and 91.7% cytotoxin [26]. In this study, the cytotoxicity of the *A. hydrophila* isolate was assessed according to the method previously described [21,26], and the cytotoxic potential of the isolate was demonstrated in BHK and Vero cells.

In vitro culture of *A. hydrophila* with the norepinephrine and epinephrine stress hormones induced marked bacterial growth, and administration of norepinephrine induced an increase in the growth and virulence of *A. hydrophila*, according to a previous study [12]. The increased bacterial growth may alter the host microenvironment, causing stress to the animal [7,8,17]. Bacteria exposed to norepinephrine have increased their ability to infect the host, and the catecholamine has the similar effects on birds [27]. Since the small intestine is richly innervated by noradrenergic nerve fibers and because the secretion of norepinephrine by sympathetic nerve fibers increases under stressful conditions, this response can affect host susceptibility to disease [12].

An important aspect of this study is the verification of the effect of stress conditions on *A. hydrophila* infection. *A. hydrophila*-infected mice treated with stress hormones required early euthanasia, as did mice belonging to the fasting subgroup, unlike mice treated with other physical stresses (low temperature and agitation). Thus, starvation may cause more severe stress to animals compared to other physical stresses, including low temperature and agitation. In this study, *A. hydrophila* was not isolated from the surviving mice subjected to epinephrine but was isolated from mice that died following epinephrine treatment. In addition, when *A. hydrophila* was serially passaged, virulence was enhanced, especially during the third passage. These data suggest that catecholamines may lead stress to animals, and an opportunistic bacterium, *A. hydrophila,* can cause disease in animals with stress.

Krzyminska et al. [28] reported the enhanced virulence of *A. caviae* isolated from the diarrheal feces of children by serial passaging in mice, with increases in virulence factors including adhesion, siderophores, and cholera-like toxin. In addition, environmental strains of *A. caviae* could be induced to produce aerolysin with three repeated passages in rabbit ileal loops [2,11]. In another study, environmental strains of aeromonads were similarly induced to produce aerolysin with repeated passaging, becoming potentially enterotoxigenic after 1–3 passages in rabbit ileal loops [29]. Presently, when *A. hydrophila* isolated from the dead penguin was inoculated by three serial passages in mice via the oral and intraperitoneal routes, the mortality of mice dramatically increased with each passage number, especially in the third passage. 

Animals caught and held in captivity for short or long periods show clinical signs similar to those of other captive zoo animals [30]. Furthermore, clinical signs and abnormal behavior tend to become exacerbated with increasing time in captivity [31]. These findings indicate that the diseases observed in captive and wild-caught animals are linked in some way to the captive environment [30]. It is likely that captivity is stressful to animals and that stress is a predisposing factor to poor health and productivity loss during the state of captivity [32]. In this study, stress may not be a direct cause of the death of penguin, but *Aeromonas* was on the only bacterium isolated from the dead penguin, indicating there is an association between death of penguin and *Aeromonas* infection. Moreover, the immune function of the stressed animals was not assessed in this study, therefore, further studies are needed to determine molecular differences between the *A. hydrophila* isolated from the penguin, and the *A. hydrophila* recovered from the mice, particularly after passaging, and to examine the immune function of the animals after inducing stress.

## 5. Conclusions

In conclusion, *A. hydrophila*, which is an opportunistic zoonotic bacterium, has the ability to infect animals with injuries involving the condition of the aquatic environments. Factors including poor sanitation and water quality, stress, overcrowding, and rough handling can make animals more sensitive to infections and trigger outbreaks of *A. hydrophila*. Here, we examined the virulence of *A. hydrophila,* previously isolated from an African black-footed penguin that died while in captivity at a zoo, and confirmed that serial passaging in mice enhanced the virulence of *A. hydrophila. Moreover*, *A. hydrophila* infection combined with administration of stress hormones or fasting increased mortality of mice.

## Figures and Tables

**Table 1:** Hemolytic activity and cytotoxicity titers of *Aeromonas hydrophila* cultured in Vero and baby hamster kidney (BHK) cells.

Strain	Rabbit Erythrocytes	Vero Cells			BHK Cells		
		0 h	3 h	24 h	0 h	3 h	24 h
Isolate	64	0	32	256	0	32	512
Reference ^1^	256	0	16	256	0	16	256
PBS	0	0	0	0	0	0	0

^1^ KCTC #2358.

**Table 2:** Mortality rate of stress-induced mice experimentally infected with *Aeromonas hydrophila*.

	No. of Dead Mice/No. of Mice Alive at the Day after Treatment				
Group *	Day 0	Day 1	Day 2	Day 3	Day 4
AH + N	0/10	6/4	4/0	-	-
AH + P	0/10	2/8	0/8	2/6	0/6
AH + F	0/10	0/10	0/10	4/6	6/0
AH + L	0/10	0/10	0/10	0/10	0/10
AH + S	0/10	0/10	0/10	0/10	0/10
AH alone	0/10	0/10	0/10	0/10	0/10
PBS	0/10	0/10	0/10	0/10	0/10

* Abbreviations: AH, *Aeromonas hydrophila*; N, norepinephrine; P, epinephrine; F, fasting; L, low temperature; S, agitation; and PBS, phosphate buffered saline.

**Table 3:** Mortality of mice experimentally infected with *Aeromonas hydrophila* according to the administration route after serial passaging.

Passage Number	Route of Inoculation	No. of Dead Mice/No. of Mice Alive Post Infection				
		0 h	12 h	24 h	48 h	72 h
1st	Oral	0/10	0/10	0/10	0/10	1/9
	Intraperitoneal	0/10	0/10	8/2	2/0	-
	PBS *	0/10	0/10	0/10	0/10	0/10
2nd	Oral	0/10	0/10	0/10	1/9	1/8
	Intraperitoneal	0/10	2/8	8/0	-	-
	PBS	0/10	0/10	0/10	0/10	0/10
3rd	Oral	0/10	10/0	-	-	-
	Intraperitoneal	0/10	10/0	-	-	-
	PBS	0/10	0/10	0/10	0/10	0/10

* PBS, phosphate buffered saline.

## Data Availability

Data presented in this study are available within the article.
